# Ultrasound Elastographic Findings of Mammary Fibromatosis

**DOI:** 10.1155/2015/829468

**Published:** 2015-02-03

**Authors:** Ping He, Li-Gang Cui, Yu-Tao Lei, Jian-Ying Liu, Jin-Rui Wang

**Affiliations:** ^1^Department of Ultrasound, Peking University Third Hospital, 49 North Garden Road, Haidian District, Beijing 100191, China; ^2^Department of Breast Surgery, Peking University Third Hospital, 49 North Garden Road, Haidian District, Beijing 100191, China; ^3^Department of Pathology, Peking University Third Hospital, 49 North Garden Road, Haidian District, Beijing 100191, China

## Abstract

Mammary fibromatosis is an uncommon, benign tumor of the breast. It is locally aggressive and has a high rate of recurrence. Its clinical presentation and imaging results always call for suspicion of malignancy. Here we describe a case of mammary fibromatosis with clinical manifestation, radiographic and pathologic results, and imaging findings from ultrasound elastography.

## 1. Introduction

Mammary fibromatosis is a rare and locally aggressive benign tumor of the breast. It originates from fibroblasts and myofibroblasts within the breast parenchyma and does not metastasize. It often occurs as an extension of lesions arising from the pectoral fascia [1]. Mammary fibromatosis accounts for less than 0.2% of all breast lesions [2]. Due to its infiltrative nature, mammary fibromatosis has a high rate of recurrence (ranging from 21% to 27% [3–5]) after inadequate surgical excision. It is difficult to distinguish mammary fibromatosis from malignant breast tumors by physical examination and imaging techniques. Here we present a mammary fibromatosis case with clinical manifestations, radiographic and pathologic features, and especially the imaging findings of ultrasound elastography.

## 2. Case Report

**Figure 1:**
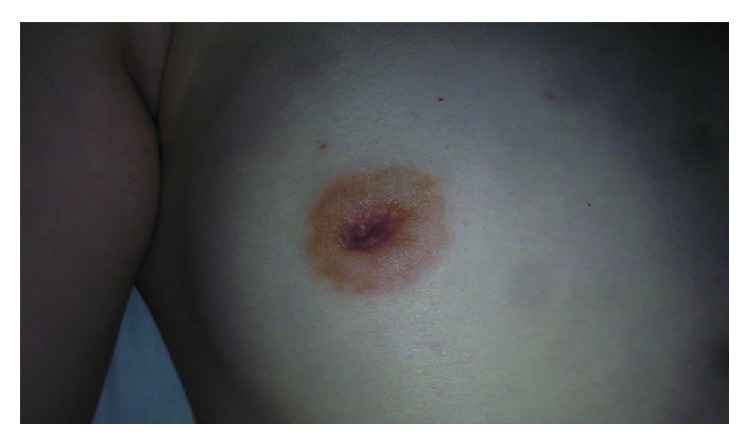
The nipple retraction was present, but there was no tenderness, redness, swelling, skin rupture, or nipple discharge.

The patient was a 22-year-old female with a 2-month history of a palpable mass in the upper outer quadrant of the right breast and nipple retraction. The size of the lesion did not change during the 2 months, and the patient claimed no other associated symptoms. On clinical examination, the nipple retracted (Figure 1), and a 3.0 × 2.0 cm firm, ill-defined, mobile mass was noted in the upper outer quadrant of the right breast with no tenderness or nipple discharge.

**Figure 2:**
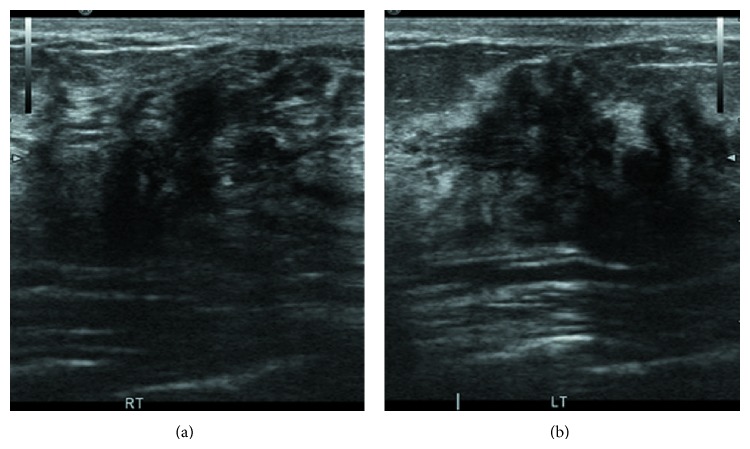
The ultrasound examination demonstrated a solid hypoechoic mass with an irregular shape and a lobulated margin both in the upper outer quadrant of the right breast (a) and below the nipple of the left breast (b).

**Figure 3:**
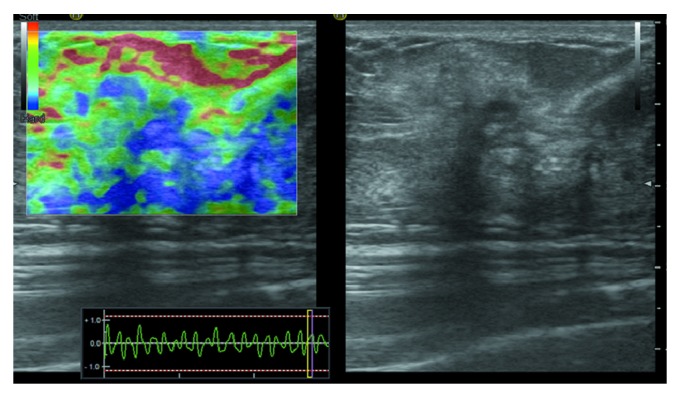
Ultrasound elastographic imaging showed very low strain value in the tumor and the surrounding area, scored as ES 5 based on the 5-point scoring system.

**Figure 4:**
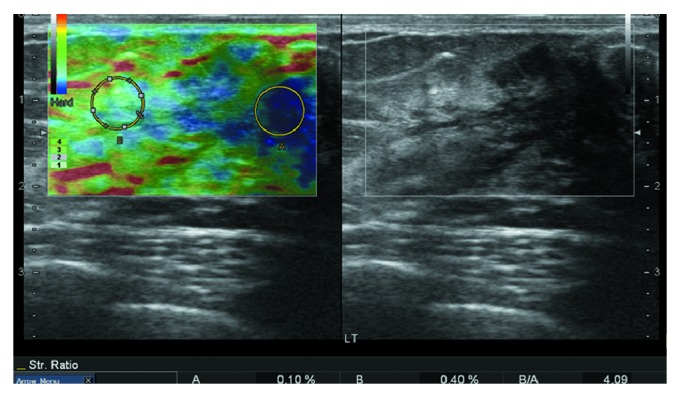
The strain ratio (the normal breast parenchyma versus the tumor) was more than 3.05.

The ultrasound examination (HI VISION Preirus) showed a solid hypoechoic mass with irregular shape and lobulated margin in the upper outer quadrant of the right breast and a second mass below the nipple of the left breast (Figure 2). No blood flow was found in the lesions. Ultrasound elastographic imaging showed very low strain value in the lesion and in the surrounding area. An elasticity score (ES) 5 (Figure 3) was given according to the 5-point scoring system [6]. The strain ratio (the normal breast parenchyma versus the lesion) was more than 3.05 (Figure 4), which suggested suspicion for malignancy and a biopsy was recommended.

Mammography showed a 3.0 × 2.5 cm mass in the right breast which was classified as BI-RADS 4 according to the Breast Imaging Reporting and Data System, and a nodule in the left breast was classified as BI-RADS 3.

An ultrasound guided biopsy was performed but the results were indeterminate and initially interpreted as hyperplasia of interstitial tissue. The patient then underwent partial mastectomy of the right breast. Gross pathologic examination of the specimen showed a 5.0 × 3.0 × 3.0 cm firm mass with nonuniform cut surfaces and ill-defined margins. The periphery of the lesion had some finger-shaped stellate extensions growing into the surrounding fat and breast tissue.

**Figure 5:**
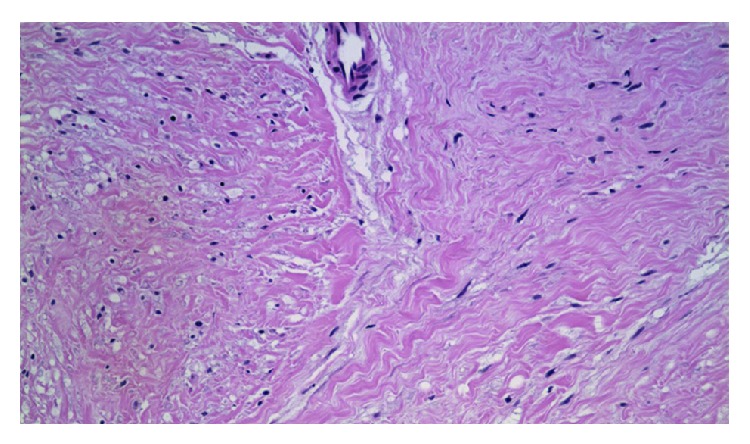
Microscopic pathological findings revealed that the lesion was composed of a spindle cell proliferation that was arranged in interlacing fascicles and formed multiple nodules with a moderate amount of collagen.

Microscopic pathologic findings revealed that the lesion was composed of spindle cell proliferations that were arranged in interlacing fascicles and formed multiple nodules with a moderate amount of collagen (Figure 5). Some residual ducts and lobules were observed within and between the nodules, but they did not form foliation. Cellular atypia was not obvious and mitotic figures were uncommon.

Immunohistochemical staining results showed that the spindle cells were positive for *β*-catenin, SMA, and VIMENTIN but were negative for DESMIN, S-100, and CK. The histology and immunohistochemical staining results supported the diagnosis of mammary fibromatosis.

## 3. Discussion

Mammary fibromatosis primarily affects females with an age range between 13 and 80 years (average age 46, median age 40), but it is more common in the childbearing age group than the perimenopausal and postmenopausal groups [1]. A few cases have also been reported in males [7]. Bilateral mammary fibromatosis has rarely been reported, with most cases occurring synchronously except for one case in which the lesions appeared asynchronously with a 2-year interval in between [8].

Mammary fibromatosis is usually painless and the presenting symptom is always a palpable, firm breast mass. Skin dimpling and nipple retraction may be present. Nipple discharge is uncommon. The etiology of mammary fibromatosis is unknown. Some cases occur after trauma or surgical procedures such as breast reduction or breast augmentation with saline or silicone implants [9]. However, it can also happen in patients with familial adenomatous polyposis (FAP) syndrome, Gardner syndrome, or hereditary desmoid diseases such as familial multicentric fibromatosis [8]. Mutations in the adenomatous polyposis coli (APC) and *β*-catenin pathway play an important role in the pathogenesis of mammary fibromatosis [8].

The reported lesion size mammary fibromatosis ranges from 0.5 to 10 cm (average 2.5–3.0 cm), and the tumor is always ill-defined with a firm, white-grey, or tan cut surface. Well-circumscribed cases are occasionally seen. On ultrasound images, it typically appears as an ill-circumscribed, lobulated, irregular, and solid hypoechoic mass with straightening and tethering of Cooper ligaments, which imitates malignant tumors. Because of its infiltrative growth pattern, the pectoralis major or intercostal muscles may be involved. However, unlike breast cancer, mammary fibromatosis does not have acoustic shadowing, echogenic halo, or microcalcification, and its orientation is usually parallel.

Ultrasound elastography is an imaging technique that can measure the stiffness of the soft tissue. It can be used to differentiate between benign and malignant breast lesions based on the principle that the stiffness of different tissues at different pathological states follows a general rule: normal fat < normal glandular < fibrous tissue < breast carcinoma. The elastographic images of breast lesions are usually scored by a 5-point scoring system described by Zhu et al. [6]. Benign lesions tend to have an ES of 1 or 2, whereas most malignant lesions have an ES of 4 or 5. A lesion with an ES of 3 could be either benign or malignant. Zhi et al. [10] suggested that the strain ratio measurement could be used for evaluating the hardness or stiffness of breast lesions semiquantitatively. When the cutoff value of the strain ratio was set at 3.05, ultrasound elastography showed a sensitivity of 92.4%, a specificity of 91.1%, and an accuracy of 91.4%. However, in the mammary fibromatosis case we reported here that both ES value (5) and strain ratio (>3.05) indicated malignant tumor, which was overruled by the final pathological results. This suggests that the ultrasound elastography may not be an ideal method to discriminate between mammary fibromatosis and malignant tumors in the breast, because the composition of mammary fibromatosis lesion makes it stiffer than normal breast tissues and may lead to a false diagnosis of malignant tumor based on the elastographic results.

Mammographically, mammary fibromatosis appears as a spiculated mass without microcalcifications which may be assessed as BI-RADS 3, 4, or 5. MRI is the best way to evaluate tumor extent and the involvement of the chest wall.

The diagnosis of mammary fibromatosis can be made from the microscopic findings on routine hematoxylin and eosin stained sections. In general, the lesion does not have malignancy features such as high mitotic rate, cellular atypia, necrosis, or vascular invasion. Lymphocytic infiltrates were often noted at the periphery of the lesion.

Since there are no specific immunomarkers for the mammary fibromatosis, immunohistochemical staining is not required for making the final diagnosis.

To prevent or reduce the recurrence, the recommended treatment for mammary fibromatosis is wide local resection.
